# Supplementary material for: Conditional chemoconnectomics (cCCTomics) as a strategy for efficient and conditional targeting of chemical transmission
Source: eLife. 2024 Apr 30;12:RP91927. doi: 10.7554/eLife.91927 (PMC11060718; doi:10.7554/eLife.91927)
Supplement: Figure 3—source data 3. [file elife-91927-fig3-data3.zip › Figure 3 source data3 part 1.zip!/Figure 3-Source data 3-part1/1081-1-CRISPResso_Report_8zosk6yj.zip!/CRISPResso_on_1081_4/9.Alleles_frequency_table_around_sgRNA_tttctggactacagtcccca.pdf]

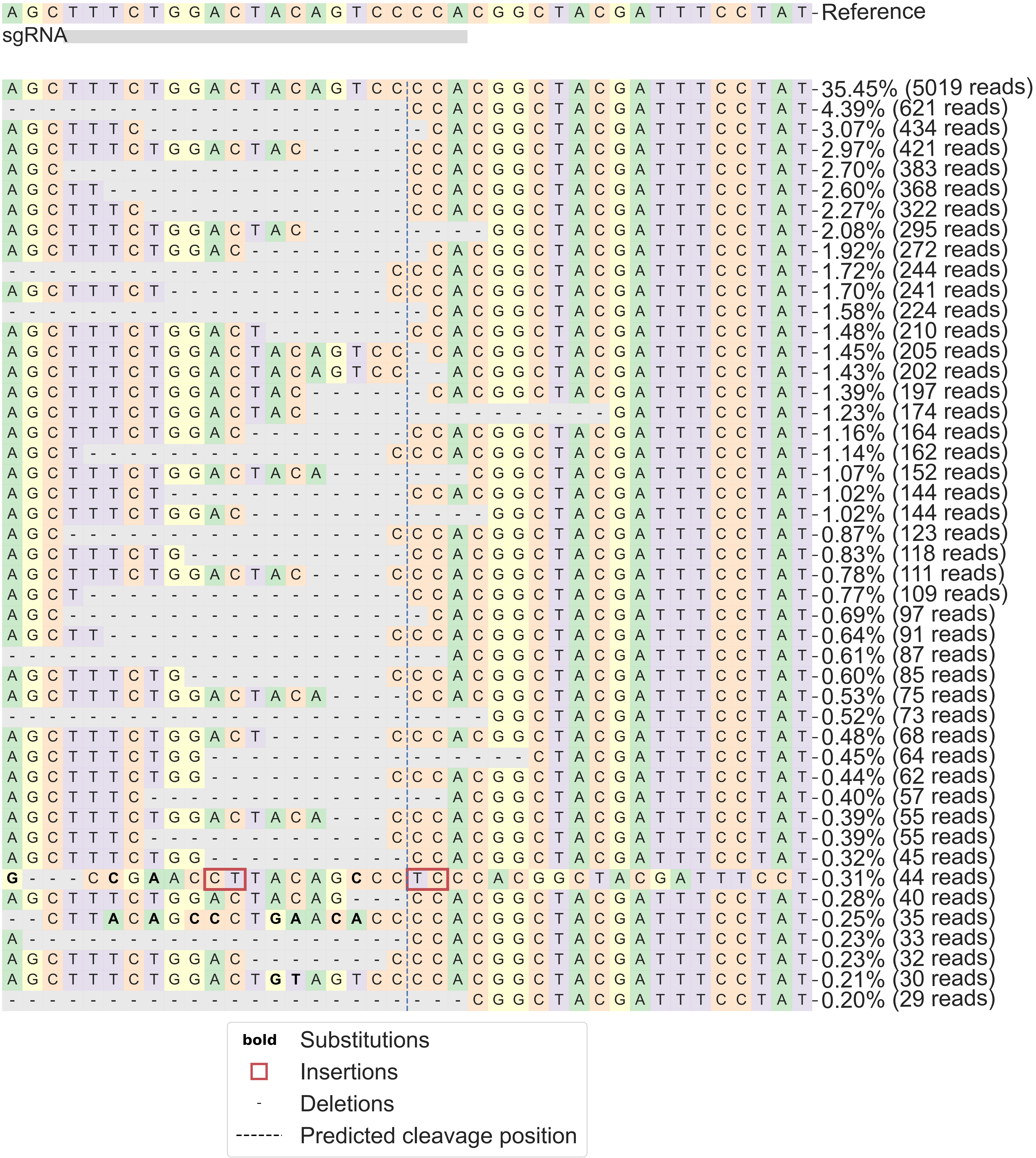
Reference
sgRNA
35.45% (5019 reads)
4.39% (621 reads)
3.07% (434 reads)
2.97% (421 reads)
2.70% (383 reads)
2.60% (368 reads)
2.27% (322 reads)
2.08% (295 reads)
1.92% (272 reads)
1.72% (244 reads)
1.70% (241 reads)
1.58% (224 reads)
1.48% (210 reads)
1.45% (205 reads)
1.43% (202 reads)
1.39% (197 reads)
1.23% (174 reads)
1.16% (164 reads)
1.14% (162 reads)
1.07% (152 reads)
1.02% (144 reads)
1.02% (144 reads)
0.87% (123 reads)
0.83% (118 reads)
0.78% (111 reads)
0.77% (109 reads)
0.69% (97 reads)
0.64% (91 reads)
0.61% (87 reads)
0.60% (85 reads)
0.53% (75 reads)
0.52% (73 reads)
0.48% (68 reads)
0.45% (64 reads)
0.44% (62 reads)
0.40% (57 reads)
0.39% (55 reads)
0.39% (55 reads)
0.32% (45 reads)
0.31% (44 reads)
0.28% (40 reads)
0.25% (35 reads)
0.23% (33 reads)
0.23% (32 reads)
0.21% (30 reads)
0.20% (29 reads)
bold Substitutions
Insertions
Deletions
Predicted cleavage position
